# Surgical Disease Burden, Outcomes, and Roles of Non-Physician Clinicians in Ugandan Emergency Departments

**DOI:** 10.5811/westjem.24989

**Published:** 2025-07-12

**Authors:** Stacey Chamberlain, Pearl Ugwu-Dike, Ronald Mbiine, Thomas Sims, Brian T Rice, Global Emergency Care Investigator Group

**Affiliations:** * University of Illinois at Chicago, Department of Emergency Medicine, Chicago, Illinois; † University of Illinois at Chicago, Center for Global Health, Chicago, Illinois; ‡ New York University, Department of Dermatology, New York, New York; § Makerere University College of Health Sciences, Department of Surgery, Kampala, Uganda; ¶ Global Emergency Care, Inc; || University of Illinois at Chicago, Department of Surgery, Chicago, Illinois; # Stanford University, Department of Emergency Medicine, Stanford, California

## Abstract

**Background:**

Delivery of emergency surgical care remains a challenge in much of Sub-Saharan Africa, with physician shortages in Uganda resulting in only one surgeon per 100,000 people. Emergency units in Uganda receive emergency surgical patients, but it is unknown how great of a burden these emergency surgical patients represent in terms of total number, care required, or outcomes.

**Methods:**

We performed a retrospective review of a quality assurance database for all patients treated at two emergency units in Uganda from 2009–2019. Patients were defined as “surgical” if they were admitted directly to the operating theatre, received a surgical diagnosis, or received an emergency surgical procedure as identified by the Disease Control Priorities 3 (DCP3) group. We generated descriptive statistics.

**Results:**

Of the 109,999 total patients seen, 24,745 (22.5%) were emergency surgical patients. Surgical patients were predominantly male (71.7%) with a mean age of 34.9 years. Most surgical patients (57.0%) were admitted to the hospital, while 38.9% were discharged, and only 1.7% were sent directly to the operating theatre. In total, 12.1% of all patients seen in the emergency unit received a surgical procedure from a non-physician clinician while in the unit. Of the surgical procedures, the most common were suturing of lacerations (51.8%), urinary catheterization (24.5%), fracture management (16.5%), and incision and drainage of abscesses (6.0%). Among surgical patients, the most common surgical diagnoses were for fractures (30.9%), lacerations (29.6%), and abscesses (8.8%). The overall three-day mortality for emergency surgical patients was 2.8%.

**Conclusion:**

Emergency surgical patients are common in Ugandan emergency units, where emergent surgical procedures are commonly performed by non-physician clinicians. Strengthening system capacity for emergency surgical patients should also consider emergency unit resources.

## INTRODUCTION

Over the last decade, inequities in global access to surgical care have gained significant attention as a public health crisis and significant contributor to high rates of preventable mortality in low- and middle-income countries (LMIC).^1^–^5^ With numerous global initiatives underway focusing on strengthening surgical systems, we need a greater understanding of existing emergency surgical disease burden, resources required, and short-term strategies to effectively care for patients where surgeons are lacking, including use of non-physician clinicians.^6^–^13^ In this study we aimed to fill some of that knowledge gap by investigating the burden of emergency surgical conditions and capacity for emergency surgical care delivery in rural Uganda.

Worldwide, five billion people lack access to essential surgical care, with the overwhelming burden of surgical disease disproportionately impacting LMICs.^1^,^3^ Although LMICs constitute 35% of the world’s population, they receive only an estimated 3.5% of all surgical interventions.^4^ In Uganda in particular, physician shortages result in only slightly more than one surgeon per 100,000 people vs 9.4 in LMICs overall and 71.2 in high-income countries.^14^ A recent study in Uganda found that less than 25% of the population had access to a surgically capable facility within two hours.^15^ Where surgical capabilities did exist, the annual surgical volume was 144.5 cases per 100,000 per year.^15^ With current rates of scale-up (5.1% per year), the target of 5,000 surgical procedures per 100,000 population per year is not projected to be achieved in Uganda until 2053.^16^

As a start to addressing these disparities, 44 surgical procedures were identified in the first volume of the *Disease Control Priorities*, 3rd edition series (DCP3), titled “Essential Surgery.” These procedures “address substantial needs, are cost effective, and are feasible to implement in [LMICs]. If made universally available, the provision of these 44 procedures would avert 1.5 million deaths a year and rank among the most cost effective of all health interventions.”^17^ Of the 44 procedures, 37 were designated as appropriate for “primary health centres” or “first-level hospitals,” and of those, 28 are designated as emergency procedures (see Table 1). Essential Surgery also suggests that non-physician clinicians can play an important role in the problem of access to basic surgery, yet this has not been studied for management of emergency surgical conditions in rural Uganda.^17^ To better understand the burden of emergency surgical conditions and capacity for emergency surgical care delivery in rural Uganda, we set out to describe the diagnoses, procedures, and outcomes associated with emergency surgical patients in two emergency units in Uganda from 2009–2019. This information will inform policy on prioritization of emergency surgical care in rural Uganda and development of systems at the intersection of emergency care and surgical care, including workforce utilization.

## METHODS

### Study Locations

We performed a retrospective review of a quality assurance database for all patients treated at two emergency units in rural southwest Uganda from November 2009–December 2019. Masaka Regional Referral Hospital is a regional referral hospital with an emergency unit patient volume of approximately 1,000 per month. Karoli Lwanga Hospital is a district-level non-governmental hospital with an emergency unit monthly patient volume of approximately 500 patients. Study sites had emergency units staffed by non-physician clinicians trained as emergency clinicians by a US and Uganda-based non-profit organization, Global Emergency Care (GEC). The non-physician clinicians were nurses who completed a two-year advanced training course in emergency care described in detail elsewhere.^18^ Study sites had limited resources with variable access to plain radiographs, no computed tomography (CT), and blood banks with inconsistent supplies of blood products. A complete description of setting, resource availability, and outcomes of the training program are also described in previous publications.^19^–^21^

### Data Collection

Beginning in 2009, GEC created a quality assurance (QA) database of emergency unit patient records to monitor and evaluate the quality of healthcare services provided at two emergency units where GEC had provided a clinical training program. Data were abstracted by trained research assistants from all consecutive emergency patients’ paper charts once the treating clinician completed the patient’s care and entered the information electronically into the database using Microsoft Excel from November 2009–March 2012 and Microsoft Access (Microsoft Corporation, Redmond, WA) from March–December 2019. Variables included patient demographics, vital signs, chief complaint, testing results, radiology results, procedures completed, medications administered, diagnoses, disposition, and three-day follow-up. All data were hand-written and transcribed verbatim when entered electronically. Three-day mortality follow-up data for emergency unit visits were collected in person for admitted patients and by structured telephone interview for patients who were discharged before three days. If a patient could not be reached on the initial attempt, calls were made daily for seven consecutive days before they were labeled as lost to follow-up. Three-day follow-up was chosen both to minimize loss to follow-up in a setting where most patients do not have consistent ability to receive phone calls and because follow-up after three days was thought to be less reflective of outcomes related to acute care provided in the emergency unit.

There were three small gaps in follow-up data collection during the study period, when no RA was available. These gaps include September 24–28, 2010; January 17–February 3, 2011; and February 13–27, 2011. Data missingness was variable throughout the 10-year, real-world implementation of this QA database. Routinely collected emergency unit data was recorded at a very high rate: sex (99.8%); age (99.3%); disposition (99.7%); and diagnosis (99.1%). Rates of completeness were lower for data about three-day mortality, which relied on data collection outside the emergency unit including in-person follow-up for admitted patients (91.0%) and phone follow-up for discharged patients (54.6%).

Inclusion criteria for this study were surgical patients of all ages seen from November 2009– December 2019. There were no exclusion criteria. Patients were defined as “surgical patients” for subsequent analysis if they met any one or more of the following criteria: 1) were admitted directly to the operating theatre; 2) received a surgical diagnosis as defined below;or 3) underwent any one of 28 emergency surgical procedures identified by the DCP3 as an essential surgical procedure for first-level hospitals and primary health centres (see Table 1).^17^ Surgical diagnoses were defined as conditions and diagnoses that would be considered indications for performance of the DCP3 procedures as identified by our study authors (see Appendix 1). Patients with more than one visit during the study period had each unique visit included in the analysis.

**Population Health Research Capsule**

What do we already know about this issue?

*There is a need for emergency surgical care in resource-limited settings (RLS)*.

What was the research question?

*What is the burden of emergency surgical disease and role of non-physician clinicians in emergency surgical care in rural Uganda?*

What was the major finding of the study?

*Of 109,999 patients, 22.5% were emergency surgical patients, and 12.1% received a procedure from a non-physician clinician*.

How does this improve population health?

*Strengthening emergency medical and surgical systems in RLS should be considered in tandem. Task sharing can be critical to improving access to emergency surgical services*.

### Data Analysis

Data was stored and processed on encrypted,. *Stata Statistical Software: Release 16* (StataCorp, LLC, College Station, TX) by a single abstractor trained in applied epidemiology [BR]. Descriptive statistics were used to describe sex, age, outcomes, dispositions, diagnoses, and procedures performed. Extensive data processing using regular expressions, string recognition and other procedural and rule-based approaches was done for free-text data analysis in Stata. This coding allowed the free-text database to be restructured into a format similar to a standard electronic health record that could be queried to produce the above variables. Sample size was based on using all available records meeting the above criteria rather than an a priori power calculation. Significance testing for continuous variables used *t*-test and for categorical variables using chi-squared test. These methods comply with optimal retrospective chart review recommendations as described by Worster and Bledsoe including abstractor training, defining variables and case selection, and descriptions of the database, sampling and analysis methods.^22^

Abstractor performance was reviewed by the lead author [SC]. Interobserver reliability was not relevant given the single data abstractor, and the abstractor was aware of study objectives. There were no cases of disagreement between abstractors about case or variable definitions. Missing data for age and patient disposition is noted in the “Results” section. The development and implementation of this database received institutional review board approval from Mbarara University of Science and Technology in Mbarara, Uganda, and the Ugandan Council of Science and Technology.

## RESULTS

Of the 109,999 total patients seen, all were examined for eligibility and, ultimately, we identified 24,745 (22.5%) with confirmed eligibility as emergency surgical patients as shown in the Figure.

The greatest proportion of surgical patients were included based on receiving a surgical diagnosis (44.8%), and the smallest proportion were included based on going directly to the operating theatre (1.7%). In total, 13,670 (12.4%) patients in the emergency units received an emergency surgical procedure. Of those receiving surgical procedures, 13,259 (97.0%) were performed by non-physician clinicians while in the emergency unit. Baseline characteristics of emergency surgical and non-surgical patients are compared in Table 2. Emergency surgical patients, as compared to their non-surgical counterparts, were significantly more likely to be male, less likely to be under five years of age, and more likely to be discharged from the emergency unit (all *P*-values <.001). The three-day mortality rate for surgical emergency patients was lower than mortality for non-surgical emergency patients (2.8% vs 3.6%, *P*<0.001). Limited information was maintained about patients referred to other facilities but the majority (n=204, 66.0%) were for orthopedic care after receiving fracture diagnoses in the emergency unit. The relative frequency of the emergent procedures performed by non-physician clinicians in the emergency unit is described in Table 3. Of the emergency unit surgical procedures, the majority were suturing of lacerations, with relief of urinary obstruction and management of non-displaced fractures also accounting for a large proportion. No emergency unit procedures were performed by physician surgeons. A prior study using the same database and individual patient records evaluated a subset of patients who required physician surgeon management in an operating theatre where the most common operative interventions were laparotomy, complex laceration repair, and herniorrhaphy.^21^

The frequency of surgical diagnoses are reported separately for all surgical patients and surgical patients that died within three days in Table 4. The relative frequency of some diagnoses is similar in both groups (e.g. fractures are most prevalent at approximately 30%). Some diagnoses are more prevalent among surgical patients overall than among patients who died within three days (e.g. laceration, dislocation). In contrast, some diagnoses are relatively more prevalent among patients who died than among the overall surgical population (e.g. bowel obstruction, acute abdomen, bowel perforation).

## DISCUSSION

### Burden of Emergency Surgical Disease

The results described above are presented to address the evidence gap that exists surrounding emergency surgical care in Uganda and in LMICs more generally. We describe a set of emergency surgical patients and procedures that represent a substantial burden but who often remain under-represented by reporting systems that look at inpatient and outpatient care but omit the emergency unit. We saw that 22.5% of all patients who arrived in two emergency units met the definition of a surgical patient, underlying the enormous burden of emergency surgical disease. Of that population, 38.9% were discharged and would not have been detected by inpatient surgical surveillance. Likewise, 12.1% of all emergency unit patients received a surgical procedure while in the emergency unit from a non-physician clinician, and these would not have been seen by studies evaluating the burden of emergency surgical disease only for patients taken to the operating theatre.^21^ These findings emphasize the need for developing health systems to consider emergency unit patients as part of development aims and ensure emergency medicine development is paired with surgical reporting and registry efforts.

Looking at our data on a more granular level, there are additional public policy implications. Four of the top five surgical diagnoses identified (fracture, laceration, blunt trauma, and dislocation) are explicitly trauma related. Surgical patients were primarily young and male, supporting other recent findings in Uganda regarding injured patients.^23^ This reinforces the need for building capacity to treat traumatic injuries, as well as for the national legislative and policy efforts targeting trauma prevention (particularly in the high-risk group of young men) and road traffic injuries, of which two-thirds are motorcycle-related in Uganda.^23^

Finally, our study found that mortality from emergency surgical patients was 2.8%. This compared favorably with mortality of non-surgical patients (3.6%); however, this still represents a substantial burden of surgical disease. In comparing diagnoses of surgical patients who survived vs died at three days, the diagnoses of bowel obstruction, acute abdomen, and bowel perforation were notably more prevalent for those who died. Trauma-related diagnoses (i.e. fractures, lacerations, blunt trauma) also contributed substantially to fatal disease burden. It is likely that patients suffering fractures and lacerations had complex poly-trauma contributing to mortality. These findings support the need for improved prehospital care measures for timely hospital transport and hemorrhage control, supporting early detection and diagnostics (e.g. point-of-care ultrasound) for surgical patients, along with public health measures to address preventable trauma.

### Role of Non-Physicians in Emergency Surgical Care

In regard to management and disposition of surgical patients, we found that although a significant proportion (38.9%) of emergency surgical patients were discharged from the emergency unit, most surgical patients (57.0%) were admitted to the hospital, but only a small percentage (1.7%) needed to be taken directly to the operating theatre. This high admission rate suggests that these patients do require inpatient care and resources, yet the vast majority of surgical admissions can be managed outside the operating room, and non-physician clinicians can significantly contribute to the care of these patients.

All of the five emergent procedures expected for primary health centers, as identified by DCP3, (normal delivery, drainage of superficial abscess, resuscitation with Basic Life Support, suturing laceration, management of non-displaced fractures) can be managed by trained non-physician clinicians, as can many of the procedures that are designated as first-level hospital procedures (e.g. relief of urinary obstruction, fracture reduction, irrigation and debridement of some open fractures, drainage of septic arthritis, debridement of osteomyelitis). In Sub-Saharan Africa, at least 25 countries use non-physician clinicians to perform medical and surgical procedures.^13^ While long-term solutions to increasing the number of trained surgeons in the region are necessary, incorporating task-shifting or task-sharing models and setting priority targets for procedural training for the most common surgical procedures (sutures, urinary catheterization, and management of non-displaced fractures) for non-physician clinicians can help address the human-resource gap for emergency surgical care.^24^ Focused training of non-physicians in triage protocols, performing trauma surveys, and identifying emergent surgical conditions may also improve outcomes with more timely diagnosis and management. Training standards, accreditation, supervision, and autonomy are all important factors that may need to be tailored to specific resource and practice environments.^9^,^13^,^20^,^21^,^24^

### Role of Emergency Medicine in Surgical Care

Finally, as surgical health systems strengthening measures are considered, attention must be paid to the role of emergency clinicians not only in provision of emergency surgical procedures, but also in diagnosis and medical resuscitation of emergency surgical patients. Timely and accurate diagnosis can be critical to improving surgical outcomes by identifying the index conditions that require emergency surgical care in the operating theatre (e.g. ectopic pregnancy, bowel perforation, appendicitis, bowel obstruction, incarcerated hernia, acute cholecystitis, traumatic intra-abdominal hemorrhage, open fractures, septic arthritis).

A previous study found that 96% of emergency surgical patients dispositioned immediately to the operating theatre had diagnostic testing (including laboratory testing and imaging) in the emergency unit.^21^ This study builds upon that work identifying surgical diagnoses associated with mortality that could be identified and prioritized for surgical consultation and resuscitative measures as a bridge to definitive operative care. Using emergency clinicians can not only improve outcomes with more timely care (e.g. wound care and laceration repair that prevents infections, catheter placement to prevent acute kidney injury from urinary outlet obstruction) but also best use physician surgeon time and expertise in the operating theatre, rather than evaluating all undifferentiated patients with abdominal pain or over-burdening them with minor surgical procedures. Further investment in emergency care training for all emergency clinicians, including non-physician clinicians, is a key element to addressing the unmet need for emergency surgical care in LMICs.

## LIMITATIONS

Our study has important limitations to consider. Both units evaluated in this study saw medical and surgical emergencies, with maternal emergencies typically being triaged to separate labour and delivery wards. Given that C-sections are estimated to comprise a third of surgical volume in most resource-limited settings, omission of this category likely biases the study toward an under-representation of emergency surgical burden in Uganda.

The specific training of the non-physician clinicians at the two study sites may pose another limitation to the generalizability of non-physician care for emergency surgical patients. The non-physician clinicians in these emergency units were trained in a two-year training course that notably included procedural sedation. Therefore, emergency clinicians with less training may not have the expertise to manage some surgical cases in emergency units.

This was a retrospective data analysis, which may have incomplete data and only included emergency unit data without the benefit of longitudinal data for admitted patients. Mortality data was limited to within three days of the emergency unit visit, and specific causes of death are unknown. Three-day mortality data was collected to minimize loss to follow-up given communication barriers in rural Uganda. However, overall surgical mortality may be underestimated without knowing longer term outcomes. In addition, we cannot draw direct inferences to cause of death, as our database was limited to surgical diagnoses associated with fatalities.

Finally, database limitations under-represent the DCP3 emergency surgical procedures of “resuscitation with Basic Life Support measures” and “resuscitation with Advanced Life Support measures” as these were not categorized by clinicians in our database as “procedures.” Without taking these measures into account, the utility of non-physician clinicians for emergency surgical patients is likely understated.

## CONCLUSION

Emergency surgical patients are common in Ugandan emergency units, comprising one fifth to one quarter of all patients seen. Many *Disease Control Priorities, 3^rd^ ed*, emergency surgical procedures are performed by non-physician emergency clinicians. Almost 60% of emergency surgical patients require hospitalisation. Strengthening system capacity for emergency surgical patients should consider emergency unit resources, in particular human resources, to optimize quality care balanced with effective health system utilization. Non-physician clinicians and other emergency care clinicians can play a critical role in meeting the human resource gap required to improve emergency surgical care.

## Supplementary Information



## Figures and Tables

**Figure:**
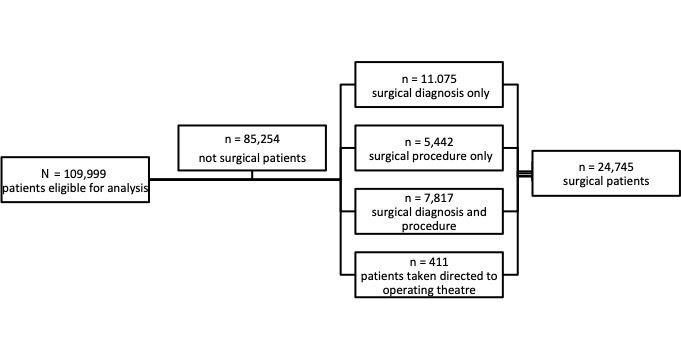
Schematic showing how emergency surgical patients were identified by category.

**Table 1:** Adapted from *Disease Control Priorities, 3^rd^ Edition*, emergency surgical procedures.

	Platform for delivery of procedure	

	Community facility and primary health centres	First-level hospitals
Obstetric, gynaecological, and family planning	Normal delivery	Caesarean birth Vacuum extraction or forceps delivery Ectopic pregnancy Manual vacuum aspiration and dilatation and curettage Hysterectomy for uterine rupture or intractable post-partum haemorrhage
General surgical	*Drainage of superficial abscess*	Repair of perforations (perforated peptic ulcer, typhoid ileal perforation, etc) Appendectomy Bowel obstruction Colostomy Gallbladder disease (including emergency surgery for acute cholecystitis) Hernia (including incarceration) *Relief of urinary obstruction; catherisation or suprapubic cytostomy (tube into bladder through skin)*
Injury	Resuscitation with Basic Life Support measures *Suturing laceration* *Management of non-displaced fractures*	Resuscitation with Advanced Life Support measures, including surgical airway *Tube thoracostomy (chest drain)* Trauma laparotomy *Fracture reduction* Irrigation and debridement of open fractures Placement of external fixator, use of traction Escharotomy or fasciotomy (cutting of constricting tissue to relieve pressure from swelling) *Trauma-related amputations* Burr hole
Non-trauma orthopedic		Drainage of septic arthritis Debridement of osteomyelitis

Note: Italicized procedures are those included in the database for this study which included emergency unit data. Procedures performed outside the emergency unit (e.g. in the operating theatre or obstetrics unit) are not registered in the emergency unit database. Additionally, resuscitative measures were not classified in this database under “procedures” and, therefore, are not represented.

**Table 2:** Baseline characteristics of emergency surgical and non-surgical patients.

	Surgical (n= 24,745)	Non-Surgical (n=85,254)	P-value
Age, mean (SD)	34.9 (23.7)	28.1 (24.2)	<.001*
Age Group, n (%)			
Under 5 years old	1,675 (6.8%)	19,548 (22.9%)	<.001
Age 5–17 years old	4,026 (16.2%)	13,503 (15.9%)	
18–44 years old	11,946 (48.3%)	31,455 (36.9%)	
45–65 years old	3,213 (13.0%)	10,892 (12.8%)	
65+ years old	3,709 (15.0%)	9,326 (10.9%)	
Age Missing	560 (0.7%)	176 (0.7%)	
Male Sex, n (%)	17,746 (71.7%)	42,910 (50.3%)	<.001
Disposition, n (%)			
Admitted	14,095 (57.0%)	56,471 (66.5%)	<.001
Discharged	9,629 (38.9%)	27,347 (32.2%)	
Expired in ED	196 (0.8%)	673 (0.8%)	
Left against medical advice or eloped	83 (0.3%)	221 (0.3%)	
Referred	309 (1.2%)	281 (0.3%)	
Sent directly to the operating theatre	411 (1.7%)	0 (0%)	
Disposition missing	22 (0.9%)	296 (0.4%)	
Mortality (Three-day), n (%)	690 (2.8%)	3,076 (3.6%)	<.001

* T-test used for significance, all other use chi-squared tests.

**Table 3:** Frequency of emergency unit surgical procedures (N=14,482 total procedures).

Procedure	Frequency of Primary Level Procedure (n,% total)	Frequency of First Level Procedure (n,% total)
Suturing laceration	7,506 (51.8%)	
Relief of urinary obstruction		3,542 (24.5%)
Management of non-displaced fracture	2,147 (14.8%)	
Drainage of superficial abscess	869 (6.0%)	
Fracture reduction		250 (1.7%)
Tube thoracostomy		87 (0.6%)
Amputation		81 (0.6%)
**Total**	**10,522 (72.7%)**	**3,960 (27.3%)**

**Table 4:** Frequency of surgical diagnoses for all patients with a surgical diagnosis and all patients with a surgical diagnosis who died.

All Patients with Surgical Diagnoses (N=19,207)		Patients with Surgical Diagnoses Who Died (n=423)	
Surgical diagnoses	Freq. (n,%)	Surgical diagnoses	Freq. (n,%)
Fracture	5,937 (30.9%)	Fracture	146 (34.5%)
Laceration	5,676 (29.6%)	Bowel obstruction	75 (17.7%)
Abscess	1,682 (8.8%)	Acute abdomen	39 (9.2%)
Blunt trauma	1,235 (6.4%)	Laceration	35 (8.3%)
Dislocation	850 (4.4%)	Blunt trauma	29 (6.9%)
Hernia	832 (4.3%)	Abscess	26 (6.2%)
Bowel obstruction	804 (4.2%)	Bowel perforation	20 (4.7%)
Urinary obstruction	532 (2.8%)	Urinary obstruction	20 (4.7%)
Acute abdomen	475 (2.5%)	Hernia	11 (2.6%)
Appendicitis	400 (2.1%)	Appendicitis	6 (1.4%)
Osteomyelitis	255 (1.3%)	Dislocation	5 (1.2%)
Bowel perforation	176 (0.9%)	Ectopic pregnancy	3 (0.7%)
Ectopic pregnancy	111 (0.6%)	Pneumothorax	3 (0.7%)
Septic arthritis	70 (0.4%)	Imperforate anus	2 (0.5%)
Pneumothorax	51 (0.3%)	Osteomyelitis	2 (0.5%)
Penetrating trauma	50 (0.3%)	Penetrating arauma	1 (0.2%)
Amputation	49 (0.3%)		
Cholecystitis	15 (0.1%)		
Imperforate anus	7 (0.04%)		
Total	19,207 (100%)	Total	423 (100%)
