# Supplementary material for: Surgical Disease Burden, Outcomes, and Roles of Non-Physician Clinicians in Ugandan Emergency Departments
Source: West J Emerg Med. 2025 Jul 12;26(4):994–1001. doi: 10.5811/westjem.24989 (PMC12342435; doi:10.5811/westjem.24989)
Supplement: Supplementary file 1 [file wjem-26-994-s001.docx]

**Appendix 1 [provided for review but not intended for publication]**

| DCP3 Emergency Surgical “Procedure” | Associated “Surgical” Diagnoses |
| --- | --- |
| normal delivery (search for “delivery”)^+^ | term pregnancy, delivery |
| drainage of superficial abscess (search for “abscess” “incision and drainage” “I&D” “I and D”+ | abscess, furuncle, pilonidal cyst |
| resuscitation with basic life support measures (search for “resuscitation”)^+^ | arrest, hemorrhage, cardiac tamponade, tension, pericardial hemorrhage |
| suturing laceration (search for “suture” “sutures” “suturing” “laceration”)^+^ | laceration |
| management for non-displaced fractures (search for “fracture” “fractures” “splint” “splinting”)^+^ | fracture |
| Caesarean birth (search for “Caesarean” or “Cesarean”) | fetal malpresentation, obstructed labour, eclampsia, pre-eclampsia, umbilical cord prolapse, placenta previa, failed induction of labour, chorioamnionitis |
| vacuum extraction or forceps delivery (search for “vacuum” or “forceps”) | obstructed labour, shoulder dystocia |
| ectopic pregnancy (search for “ectopic”) | ectopic pregnancy |
| manual vacuum aspiration and dilatation and curettage (search for “aspiration”, “dilatation”, “curettage”) | retained products of conception, post-partum hemorrhage |
| hysterectomy for uterine rupture or intractable post-partum hemorrhage (search for “hysterectomy”, “uterine rupture”, “hemorrhage”) | uterine rupture, post-partum hemorrhage |
| repair of perforations (search for “perforation”) | perforated; perforation, perforated bowel, perforated ulcer, bowel perforation, typhoid ileal perforation) |
| appendectomy (search for “appendectomy”) | appendicitis |
| bowel obstruction (search for “obstruction”) | Obstructed, obstruction, mechanical ileus, intussusception, volvulus, pyloric stenosis |
| colostomy (search for “colostomy”) | sigmoid volvulus, penetrating abdominal injury, large bowel obstruction, abdominal mass, imperforate anus, Hirschsprung’s disease, cloaca, rectal trauma |
| gallbladder disease (including emergency surgery for acute cholecystitis) (search for “cholecystitis”) | cholecystitis, cholangitis, choledocholithiasis, cholelithiasis, porcelain gallbladder, gallbladder calcification |
| hernia (including incarceration) (search for “hernia”) | incarcerated hernia, strangulated hernia |
| relief of urinary obstruction; catheterisation or suprapubic cystostomy (search for “obstruction”, “catheterisation”, “catheterization”, “cystostomy”, “suprapubic” and “catheter”) | urinary obstruction |
| resuscitation with advanced life support measures, including surgical airway (search for “cricothyroidotomy”, “tracheostomy”) | airway obstruction |
| tube thoracostomy (search for “thoracostomy” “chest tube”) | pneumothorax, hemothorax, pneumohemothorax, hemopneumothorax, flail chest |
| trauma laparotomy (search for “laparotomy”, “DPL”, “Deep Peritoneal Lavage”) | acute abdomen, surgical abdomen, abdominal trauma, penetrating abdomen, hemoperitoneum, blunt injury, diaphragm rupture, traumatic diaphragmatic hernia, diaphragm injury, evisceration |
| fracture reduction (search for “fracture” and “reduction”) | fracture |
| irrigation and debridement of open fractures (search for “open fracture”) | open fracture |
| placement of external fixator, use of traction (search for “external fixator” and “traction”) | fracture, open fracture |
| escharotomy or fasciotomy (search for “escharotomy” and “fasciotomy”) | compartment syndrome, fasciitis |
| trauma-related amputations (search for “amputation”) | amputation |
| burr hole (search for “burr hole”) | epidural hematoma, subdural hematoma, intracranial hemorrhage, herniation, Cushing’s triad |
| drainage of septic arthritis (search for “septic arthritis” and “arthrocentesis”, “drainage”) | septic arthritis |
| debridement of osteomyelitis (search for “osteomyelitis”) | osteomyelitis |

- Procedures recommended for primary health centres
